# Executive task-based brain function in children with type 1 diabetes: An observational study

**DOI:** 10.1371/journal.pmed.1002979

**Published:** 2019-12-09

**Authors:** Lara C. Foland-Ross, Bruce Buckingam, Nelly Mauras, Ana Maria Arbelaez, William V. Tamborlane, Eva Tsalikian, Allison Cato, Gabby Tong, Kimberly Englert, Paul K. Mazaika, Allan L. Reiss, for the Diabetes Research in Children Network (DirecNet)

**Affiliations:** 1 Center for Interdisciplinary Brain Sciences Research, Department of Psychiatry and Behavioral Sciences, Stanford University, Stanford, California, United States of America; 2 Division of Pediatric Endocrinology and Diabetes, Stanford University School of Medicine, Stanford, California, United States of America; 3 Division of Endocrinology, Diabetes and Metabolism, Nemours Children’s Health System, Jacksonville, Florida, United States of America; 4 Division of Endocrinology, Washington University, Saint Louis, Missouri, United States of America; 5 Division of Endocrinology, Yale University, New Haven, Connecticut, United States of America; 6 Division of Endocrinology, University of Iowa, Iowa City, Iowa, United States of America; 7 Division of Neurology, Nemours Children’s Health System, Jacksonville, Florida, United States of America; 8 Department of Pediatrics, Stanford University School of Medicine, Stanford, California, United States of America; 9 Department of Radiology, Stanford University School of Medicine, Stanford, California, United States of America; Universidad de Guadalajara, MEXICO

## Abstract

**Background:**

Optimal glycemic control is particularly difficult to achieve in children and adolescents with type 1 diabetes (T1D), yet the influence of dysglycemia on the developing brain remains poorly understood.

**Methods and findings:**

Using a large multi-site study framework, we investigated activation patterns using functional magnetic resonance imaging (fMRI) in 93 children with T1D (mean age 11.5 ± 1.8 years; 45.2% female) and 57 non-diabetic (control) children (mean age 11.8 ± 1.5 years; 50.9% female) as they performed an executive function paradigm, the go/no-go task. Children underwent scanning and cognitive and clinical assessment at 1 of 5 different sites. Group differences in activation occurring during the contrast of “no-go > go” were examined while controlling for age, sex, and scan site. Results indicated that, despite equivalent task performance between the 2 groups, children with T1D exhibited increased activation in executive control regions (e.g., dorsolateral prefrontal and supramarginal gyri; *p* = 0.010) and reduced suppression of activation in the posterior node of the default mode network (DMN; *p* = 0.006). Secondary analyses indicated associations between activation patterns and behavior and clinical disease course. Greater hyperactivation in executive control regions in the T1D group was correlated with improved task performance (as indexed by shorter response times to correct “go” trials; *r* = −0.36, 95% CI −0.53 to −0.16, *p <* 0.001) and with better parent-reported measures of executive functioning (*r* values < −0.29, 95% CIs −0.47 to −0.08, *p*-values < 0.007). Increased deficits in deactivation of the posterior DMN in the T1D group were correlated with an earlier age of T1D onset (*r* = −0.22, 95% CI −0.41 to −0.02, *p* = 0.033). Finally, exploratory analyses indicated that among children with T1D (but not control children), more severe impairments in deactivation of the DMN were associated with greater increases in hyperactivation of executive control regions (T1D: *r* = 0.284, 95% CI 0.08 to 0.46, *p* = 0.006; control: *r* = 0.108, 95% CI −0.16 to 0.36, *p* = 0.423). A limitation to this study involves glycemic effects on brain function; because blood glucose was not clamped prior to or during scanning, future studies are needed to assess the influence of acute versus chronic dysglycemia on our reported findings. In addition, the mechanisms underlying T1D-associated alterations in activation are unknown.

**Conclusions:**

These data indicate that increased recruitment of executive control areas in pediatric T1D may act to offset diabetes-related impairments in the DMN, ultimately facilitating cognitive and behavioral performance levels that are equivalent to that of non-diabetic controls. Future studies that examine whether these patterns change as a function of improved glycemic control are warranted.

## Abstract 2

Lars C. Foland Ross and colleagues investigate executive brain function in children with Type 1 diabetes.

## Author summary

**Why was this study done?:**

- Maintaining optimal levels of blood glucose in children with type 1 diabetes (T1D) is difficult.
- Because childhood and adolescence are periods of major neurodevelopmental change, there has been growing interest in whether T1D is associated with variations in the brain.
- No studies to date, to our knowledge, have examined brain function in children with T1D as they engaged in a cognitively demanding task.

**What did the researchers do and find?:**

- Brain function was examined in 150 children with or without T1D as they performed an executive function paradigm, the go/no-go task. This task requires that participants respond as quickly and accurately as possible, using a button press, to a high number of “go” stimuli (e.g., when they see any letter except “X”), and suppress a prepotent response on a smaller subset of “no-go” stimuli (e.g., when they see the letter “X”).
- Despite equivalent performance on the go/no-go task, children with T1D exhibited increased activation in task-positive networks (e.g., in executive control regions), as well as reduced suppression of activation in the default mode network (DMN).
- Increased hyperactivation in task-positive networks was associated with better executive functioning, improved go/no-go task performance, and reduced suppression of the DMN in children with T1D.
- Reduced suppression of the DMN, in turn, was associated with an earlier age of onset of T1D.

**What do these findings mean?:**

- These data indicate that increased recruitment of executive control areas in pediatric T1D may act to offset diabetes-related impairments in the DMN and cognitive function.
- Future studies that examine whether these patterns change as a function of improved glycemic control are warranted.

## Introduction

Type 1 diabetes (T1D) is the second most prevalent chronic health condition affecting children under the age of 18 years [1,2]. Despite careful monitoring of insulin dosing, diet, and exercise, most children with T1D have difficulty maintaining optimal levels of blood glucose. The question of whether dysglycemia (i.e., abnormal blood glucose levels, which may be characterized as hypoglycemia and hyperglycemia) has an effect on the developing brain—one of the body’s most metabolically active organs—has attracted the attention of both the diabetes and neuroscience research communities. Because childhood and adolescence are periods of major neurodevelopmental change [3,4], disruptions in glucose metabolism during these sensitive developmental periods may have lasting effects on brain development and associated cognition.

Consistent with this notion, several studies [5,6], including research from our group [7–13], have observed altered brain structure in children with T1D relative to age- and sex-matched healthy controls. Cross-sectional investigations indicate that these changes originate shortly after diagnosis and last well into adulthood [14,15]. Whether morphological changes in the brain contribute to the mild disruptions in cognitive function that have been documented in this population is unclear. Children and adolescents with T1D, for example, are more likely to perform somewhat worse on tasks that require sustained attention, rapid processing speed, memory, and visuospatial functioning compared to their non-diabetic peers [16,17]. Recent meta-analyses show that executive functions, including processes like working memory, attention, and response inhibition, are particularly affected [18].

Given the structural alterations in the brain that have been documented in youth with T1D, it may be considered surprising that children living with this condition do not experience more significant cognitive impairment. One mechanism that may act to preserve normal neurocognitive function in the face of altered brain structure is a compensatory increase in brain activation. Studies of adults with T1D, for example, consistently find diabetes-related hyperactivation in executive control regions during the performance of cognitively demanding tasks [19–21]. A hypothesis of compensatory brain function may also account for observations of increased resting state functional connectivity (RSFC) in adults with T1D [22–24]. Interestingly, prior work has demonstrated that individuals with T1D scanned after the onset of microangiopathy show no such increase, suggesting that compensatory elevations in brain function may be a finite phenomenon that is eventually lost with disease progression [22]. Moreover, T1D and healthy control groups across many of these studies do not differ with respect to overall cognitive function. Yet high activation and connectivity estimates are consistently correlated in the T1D group with improvements in executive function. These findings, taken together, suggest increased brain activation in T1D may transiently counteract the negative influences of this condition on brain structure and cognition.

In the current study, we sought to extend these findings through examining—for the first time, to our knowledge—brain function in children with T1D as they engaged in a cognitive challenge. Specifically, we scanned 150 children as they performed the classic executive function/response inhibition paradigm, the go/no-go task [25,26]. Widely used to assess attention and inhibition difficulties in a clinical setting, this task requires that participants respond as quickly and accurately as possible, using a button press, to a high number of “go” stimuli (e.g., when they see any letter except “X”), and suppress a prepotent response on a smaller subset of “no-go” stimuli (e.g., when they see the letter “X”). We hypothesized on the basis of prior work in adults, that children with T1D would perform the task as well as non-diabetic (control) children, but would exhibit anomalous increases in activation of task-relevant brain regions, such as the inferior frontal gyrus, dorsal anterior cingulate cortex, and posterior parietal cortex. We additionally examined in secondary analyses whether T1D-related alterations in brain activation shared associations with measures of behavior, cognitive function, dysglycemia, and disease course.

## Methods

### Participants

The study was approved by the institutional review boards at each participating research center and an National Institutes of Health–designated data and safety monitoring board. All parents or legal guardians provided written informed consent, and participants provided assent, according to local guidelines. Participants between 7 and 14 years of age were recruited from and underwent cognitive, glycemic, and neuroimaging data collection procedures at 1 of 5 US clinical centers participating in the Diabetes Research in Children Network (DirecNet) study group (Nemours Children’s Health System in Jacksonville, Florida; Stanford University in Stanford, California; University of Iowa in Iowa City, Iowa; Washington University in St. Louis, Missouri; and Yale University in New Haven, Connecticut). The Nemours Children’s Health System in Jacksonville served as the clinical coordinating center for the study. The Stanford Center for Interdisciplinary Brain Sciences Research served as the imaging and data coordinating center (IDCC) for the study, and research personnel from the IDCC directed all image processing and analyses. Participants in the T1D group were diagnosed at ≥6 months of age and used insulin for at least 1 month prior to study enrollment. Family reports of a prior history of diabetic ketoacidosis (DKA) were confirmed with medical records. Participants in the non-diabetic control group had glycated hemoglobin (HbA_1c_) result < 6.0% (<42 mmol/mol), fasting glucose < 110 mg/dl (<6.1 mmol/l), and no history of abnormal blood glucose. Sibling controls of participants with T1D (*N* = 8) also had negative islet cell autoantibody testing within 1 year of enrollment. Exclusion criteria for both the T1D and control groups included past medical history of disorders that could impair neurological development, intellectual disability or significant learning disabilities, psychiatric treatment, premature birth (<34 weeks gestation), low birth weight (<2,000 g), and MRI contraindications.

Study data were acquired from a total of 163 children, including 103 individuals with T1D and 60 non-diabetic control participants. Research personnel at the IDCC reviewed MRI scan and behavioral data for quality. Three scans from the control group and 10 scans from the T1D group were excluded due to poor scan quality because of motion artifacts or because the participant was not performing the task as instructed (see below for further detail on exclusionary criteria). This resulted in final group sizes of 93 individuals with T1D (mean age ± SD, 11.5 ± 1.8 years; 45.2% female) and 57 control participants (mean age ± SD, 11.8 ± 1.5 years; 50.9% female).

### Cognitive testing and blood glucose measurement

Cognitive and behavioral assessment was performed with the goal of completing these measures within 4 weeks of scanning. Parents completed the Behavior Assessment System for Children, Second Edition (BASC-2) [27], and the Behavior Rating Inventory of Executive Function (BRIEF) [28]. Children were administered portions of the Wechsler Intelligence Scale for Children, Fourth Edition (WISC-IV) [29]. The order of the testing (with respect to scanning) was not mandated. Age-normed *T* scores on BASC-2 and BRIEF subscales relevant to the construct of response inhibition (i.e., Externalizing Problems, Hyperactivity, Attention Problems, and Behavioral Symptoms Index in BASC-2 and Inhibition Problems and Global Executive Composite in BRIEF), as well as *T* scores on the Processing Speed Index in the WISC-IV, were used in analyses examining associations between cognitive ability and brain activation measures.

A hyperglycemic index was determined for participants with T1D based on all available HbA_1c_ values since diagnosis up to the time of participation in this study. As in previous studies [9,10], this index was obtained by computing the area under the curve HbA_1c_ > 6.0% (HbA_1c_AUC_6%_) according to the trapezoid rule. Other covariates of interest were computed from data acquired using a continuous glucose monitor (CGM) device that was worn with the goal of obtaining >72 hours of glycemic data (including at least 24 hours of overnight data) across 2 separate 6-day periods within 90 days of the scan. Resulting measures included mean blood glucose; 2 measures of glycemic variability, the coefficient of variation (CV) of CGM glucose and mean amplitude of glycemic excursion (MAGE); and 2 measures reflecting percentage and severity of blood glucose values in hyperglycemic and hypoglycemic ranges, area under the curve above blood glucose 180 mg/dl (10 mmol/l) (AUC180) and area over the curve below blood glucose 70 mg/dl (3.9 mmol/l) (AOCBelow70), respectively. The Jaeb Center for Health Research analyzed all glucose data and provided glycemic metrics.

### MRI acquisition

All participants were prepared for unsedated MRI scans through scan simulations performed by research staff at each of the study sites, as previously described [8]. A fingerstick blood glucose level was obtained for participants with T1D immediately before and after each scan session to ensure the glucose level was between 70 and 300 mg/dl (3.9 and 16.7 mmol/l). These 2 measurements were averaged together to compute an average blood glucose level during the time of scan. Rate of glucose change (per minute) during the MRI scan was also calculated, by dividing the change in pre- and post-scan blood glucose levels by the duration of time between the 2 fingerstick tests.

Participants were scanned at 1 of 5 imaging sites using identical Siemens 3T Tim Trio whole body MR systems with matching 12-channel head coils and imaging protocols. T1-weighted structural images of the brain were acquired sagittally, right to left, using a magnetization-prepared rapid gradient echo (MP-RAGE) sequence, with slice thickness = 1 mm, repetition time (TR) = 2,300 ms, echo time (TE) = 2.98 ms, inversion time (TI) = 900 ms, flip angle = 9°, FOV = 25.6 cm × 24 cm, 160 slices, matrix = 256 × 256, voxel size = 1 × 1 × 1 mm, and scan duration 4:54 minutes. Scans were repeated if needed, as per protocol, in order to increase the probability of obtaining a low-motion, usable scan [30]. T2*-weighted functional images of the brain were acquired during the performance of each of 2 go/no-go task runs using an echo planar imaging (EPI) sequence with TR = 2,000 ms, TE = 27 ms, flip angle = 80°, FOV = 22 cm × 22 cm, 33 slices, matrix = 74 × 74, voxel size = 3 × 3 × 4 mm, and scan duration 8:26 minutes.

### Go/no-go task design

Stimuli were presented using E-Prime, version 2 (Psychology Software Tools), using a video projector that illuminated a rear projection screen located at the end of the magnet. Participants viewed stimuli through an adjustable mirror attached to the head coil, and MRI acquisition was synchronized with the paradigm. Participants were presented with a fixed series of letters in 80-point font on the center of the screen and instructed to respond, using a response box held in their dominant hand, to every letter (go trial) with a key press, except to the letter “X” (no-go trial). Each letter trial was presented for 250 ms and was separated from the subsequent trial with a jittered intertrial interval that ranged from 750 ms to 8,750 ms, during which participants passively viewed a fixation cross. The task was weighted towards go stimuli (*N* = 300 trials) to build up a prepotent tendency to respond, thereby increasing the inhibitory effort necessary to successfully withhold responding to no-go stimuli (*N* = 75 trials). The task was divided into 2 separate runs, each lasting 8.3 min. Accuracy of responses and response times (RTs) were recorded.

### Behavioral data analyses

Statistical analyses were carried out using SPSS (version 22.0). After checking distributions with the Shapiro–Wilks test, and transforming data if necessary, group differences in task performance—including commission errors, omission errors, RT for correct go trials, and the signal detection measure, d-prime—were assessed using multivariate analysis of covariance (MANCOVA), controlling for age and sex. Significance was assessed using a 2-tailed α level of 0.05.

### Primary analyses: Group differences in activation

Preprocessing of functional MRI (fMRI) data was conducted in FSL (FMRIB Software Library), version 5.0.8, using FEAT (FMRI Expert Analysis Tool), version 6.0.0. The first 3 volumes of each scan were discarded to allow for stabilization of longitudinal magnetization. The remaining images were preprocessed using a series of steps. First, non-brain material was removed from both the anatomical and functional images using the Brain Extraction Tool [31]. Preprocessing included motion correction to the mean image [32], spatial smoothing using a Gaussian smoothing kernel of 6-mm FWHM, and high-pass temporal filtering [33]. Linear registration was performed using FMRIB’s Linear Image Registration Tool to linearly align each individual’s functional data to his/her high-resolution anatomical image [34]. Nonlinear registration was used to align each individual’s anatomical image to standardized space using a publicly available template created for children aged 7–11 years in Montreal Neurological Institute (MNI) space [35,36]. The linear and nonlinear transformations were combined to register each individual’s functional data to template space.

Because 2 separate task runs were conducted for each participant, time-series statistical analyses were carried out at a single-run intraindividual level using a generalized linear model that modeled each condition and accuracy type (go correct, go incorrect, no-go correct, no-go incorrect) using a synthetic hemodynamic response function and its first derivative, as well as motion correction parameters and time points that exceeded a motion threshold (75th percentile plus 1.5 times the interquartile range) defined by FSL’s motion outliers tool (http://fsl.fmrib.ox.ac.uk/fsl/fslwiki/FSLMotionOutliers). Both runs were combined in a fixed effects analysis for each participant to provide individual-specific summaries of activation.

To test for voxel-based group differences in activation, individual-specific activation summary maps for the no-go correct minus go correct (“no-go > go”) contrast were computed and carried to higher-level voxel-based analyses using FMRIB’s Local Analysis of Mixed Effects (FLAME) [37] to assess a main effect of group while controlling for age, sex, and scan site. Resulting statistical images were thresholded at *Z* > 3.1 and a cluster probability of *p* < 0.01, corrected for whole-brain multiple comparisons using Gaussian random-field theory [38].

In addition to voxel-based analyses, region of interest (ROI)–based analyses were conducted to target regions directly involved in response inhibition. These analyses involved 3 main steps. First, data-driven, unbiased spatial maps of activation of executive control regions were created by running the no-go > go contrast across groups, controlling for age, sex, and scan site. To reduce the bias from the fact that the number of participants in the group with T1D was twice that in the control group, we randomly selected 57 participants from the group with T1D to match the number of participants in the control group without diabetes. The 2 resulting balanced groups did not vary with respect to age, sex, or scan site. Second, using voxel-based analyses with FLAME, we tested for the main effect of condition (no-go > go), controlling for age, sex, and scan site, using a threshold of *Z* > 3.1 and a cluster probability of *p* < 0.01, corrected [38]. This resulted in a set of clusters that were subsequently isolated and binarized to create functional ROIs. Lastly, percent signal change values for the no-go > go contrast were extracted from each of the resulting ROIs for each participant from the entire study sample (*N* = 150), using FSL’s featquery tool. Percent signal change values were analyzed using MANCOVA to test for a main effect of diagnosis while controlling for age, sex, and scan site.

### Secondary analyses: Associations between activation and clinical and behavioral measures

Associations between activation and behavioral, cognitive, and clinical measures were examined using correlation analyses. After checking distributions with the Shapiro–Wilks test, associations between normally distributed variables were assessed by Pearson correlation. Data that were not normally distributed were analyzed using Spearman correlation. Activation (adjusted for age, sex, and site) was entered as the dependent variable. Behavioral, cognitive, and clinical measures (adjusted for age, sex, and site where appropriate) were entered as independent variables. To reduce the number of multiple comparisons, 2 summary metrics were computed for use in these analyses: mean percent signal change occurring across all voxels contained within significant clusters resulting from our primary (voxel-based) analyses, and mean percent signal change occurring across voxels contained within all data-driven ROIs. MAGE was additionally adjusted for mean blood glucose to discriminate between the effects of glucose variability and glucose mean. Correction for multiple comparisons across these correlations was conducted using the Hochberg step-up approach [39] using a false discovery rate of 0.1.

## Results

### Participants

The T1D and control groups did not differ with respect to age (*t*[148] = 0.996, *p* = 0.321) or sex (*χ*^2^ = 0.305, *p* = 0.616; Table 1). Eight sibling pairs discordant for T1D were included in the study sample. All findings presented below remained unchanged when unaffected siblings from these 8 pairs were removed from the analyses.

**Table 1:** Clinical characteristics of study participants.

Characteristic	T1D	Control	*p*-Value
General information			
*N* (female/male)	93 (43/50)	57 (29/28)	0.616
Age (years), mean ± SD	11.5 ± 1.8	11.8 ± 1.5	0.321
Go/no-go task performance			
Commission errors, median (25th, 75th percentile)	26 (18, 45)	31 (16, 47)	0.554
Omission errors, median (25th, 75th percentile)	20 (10, 36.5)	11 (5.5, 39.5)	0.715
RT to correct “go” trials (ms), mean ± SD	469.8 ± 67.2	462.7 ± 67.0	0.444
d-prime, mean ± SD	1.7 ± 0.8	1.9 ± 0.9	0.593
Behavioral measures (*T* scores)			
BASC-2 Externalizing Problems, median (25th, 75th percentile)	47 (41.25, 53)	46 (41.25, 51)	0.221
BASC-2 Hyperactivity, median (25th, 75th percentile)	48 (42, 55)	47 (42, 53)	0.350
BASC-2 Attention Problems, median (25th, 75th percentile)	50 (44, 56)	50 (41, 52)	0.512
BASC-2 Behavioral Symptoms Index, median (25th, 75th percentile)	48 (43, 53)	45 (42, 51)	0.342
BRIEF Inhibition Problems, median (25th, 75th percentile)	47 (42, 56.5)	49 (42.25, 55)	0.735
BRIEF Global Executive Composite, median (25th, 75th percentile)	50 (42, 56.75)	48 (42.25, 54.5)	0.449
WISC-IV Processing Speed Index, median (25th, 75th percentile)	99.5 (94, 109)	100 (85, 109)	0.186
Clinical measures			
Age at diabetes onset (years), mean ± SD	4.4 ± 2.1	—	—
HbA_1c_, median (25th, 75th percentile)	8.1 (7.5, 8.8)	5.2 (5.1, 5.4)	<0.001
Lifetime averaged HbA_1c_AUC_6%_, median (25th, 75th percentile)	12.4 (7.9, 16.9)	—	—
Blood glucose level at scan (mg/dl), median (25th, 75th percentile)	157 (121.5, 206.0)	—	—
Blood glucose rate of change during scan (mg/dl per minute), mean ± SD	−0.143 ± 0.716	—	—
CGM mean glucose (mg/dl), mean ± SD	195.0 ± 34.8	—	—
CGM glucose CV (mg/dl), mean ± SD	0.42 ± 0.06	—	—
CGM MAGE (mg/dl), mean ± SD	149.4 ± 26.6	—	—
CGM AUC180 (mg/dl), mean ± SD	44.6 ± 23.7	—	—
CGM AOCBelow70 (mg/dl), median (25th, 75th percentile)	0.5 (0.1, 1.1)	—	—
Lifetime history of seizures or loss of consciousness, *n* (%)	4 (4.3)	0 (0)	—
DKA history, *n* (%)	29 (31.2)	—	—
Severe hypoglycemia history, *n* (%)	12 (12.9)	—	—

Lifetime averaged HbA_1c_AUC_6%_ (area under the curve HbA_1c_ > 6.0) represents the lifetime hyperglycemic index based on all available HbA_1c_ values since diagnosis and up to the time of participation in this study. AOCBelow70, area over the curve below blood glucose 70 mg/dl; AUC180, area under the curve above blood glucose 180 mg/dl; BASC-2, Behavior Assessment System for Children, Second Edition; BRIEF, Behavior Rating Inventory of Executive Function; CGM, continuous glucose monitor; CV, coefficient of variation; DKA, diabetic ketoacidosis; MAGE, mean amplitude of glycemic excursion; RT, response time; SD, standard deviation; T1D, type 1 diabetes; WISC-IV, Wechsler Intelligence Scale for Children, Fourth Edition.

Within the T1D group, the mean age at TID diagnosis (also referred to as the age of TID onset) was 4.4 ± 2.1 years. The mean glucose during the scan was 166 ± 51 mg/dl. As expected, HbA_1c_ was normal in the control group (median = 5.2%), and hence significantly higher in the T1D group (median = 8.1%, *U* = 1.500, *p* < 0.001). Twelve children with T1D (12.9%) experienced 1 or more episodes of severe hypoglycemia since diagnosis; 29 had (31.2%) experienced 1 or more episodes of DKA.

### Go/no-go task performance

Commission errors, omission errors, RT, and d-prime values are presented in Table 1. Task performance did not vary as a function of group (*F*[1,142] = 0.582, *p* = 0.676). Age was a significant predictor of performance (*F*[1,142] = 17.430, *p <* 0.001). Planned follow up comparisons showed age was a significant predictor of d-prime (*F*[1,142] = 24.099, *p <* 0.001, β = 0.194), RT to correct go trials (*F*[1,142] = 12.293, *p* = 0.001, β = −10.908), number of omission errors (*F*[1,142] = 21.649, *p <* 0.001, β = −6.272), and number of comission errors (*F*[1,142] = 21.649, *p <* 0.001, β = −1.790).

### Cognitive and blood glucose measurement

Within the T1D group, an average of 277 ± 63 hours of CGM data were collected per participant. CGM measures of dysglycemia are presented in Table 1. No differences in BASC-2, BRIEF, or WISC-IV measures of attention, inhibition, executive function, or processing speed were observed between groups (*t* values[146] < |1.229|, *p*-values > 0.186).

### Voxel-based fMRI analyses

**Fig 1:**
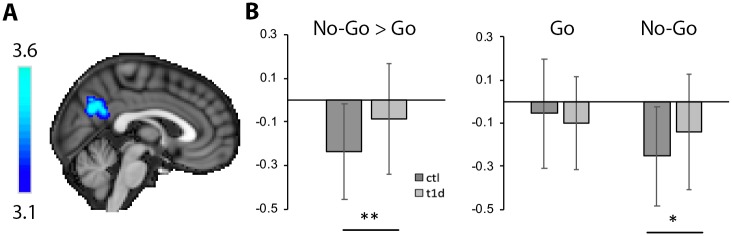
Voxel-based activation differences between groups in the posterior cingulate cortex/precuneus. (A) Statistical activation map from voxel-based analyses, showing the location of activation differences between groups during the no-go > go contrast in the posterior node of the default mode network. (B) Percent signal change from this cluster indicated greater deactivation in the control (ctl) relative to the type 1 diabetes (T1D) group. Planned pairwise comparisons showed that this finding was driven by deactivation differences occurring during no-go trials. Error bars represent standard deviation. *p < 0.01, **p < 0.001.

A significant effect of group for the contrast of no-go > go was observed in a cluster that was centered in the posterior node of the default mode network (DMN) and included parts of the posterior cingulate cortex (PCC) and precuneus (PCu; *x/y/z* peak MNI coordinates = 2, −64, 30, *k* = 2,432 voxels, *p* = 0.006; Fig 1A). Decomposition of multifactor effects indicated greater deactivation in the control group relative to the T1D group during no-go trials (*F*[1,142] = 8.119, *p* = 0.005). No difference in activation was present between groups during go trials (*F*[1,142] = 1.200, *p* = 0.275; Fig 1B; Table 2). Two-tailed 1-sample *t* tests indicated that percent signal change in the PCC/PCu cluster during no-go > go was significantly less than 0 in both groups (*t* values < −3.500, *p*-values < 0.002).

**Table 2:** Voxel-based activation differences between groups in the posterior cingulate cortex/precuneus.

Trial	Type 1 diabetes	Control	*p*-Value
No-go > go	−0.09 ± 0.25	−0.24 ± 0.22	0.006
No-go	−0.14 ± 0.27	−0.23 ± 0.23	0.005
Go	−0.10 ± 0.21	−0.06 ± 0.25	0.275

Values indicate raw percent signal change (mean ± standard deviation) extracted from a significant cluster identified in voxel-based analyses of the interaction of group by condition.

### ROI-based fMRI analyses

**Fig 2:**
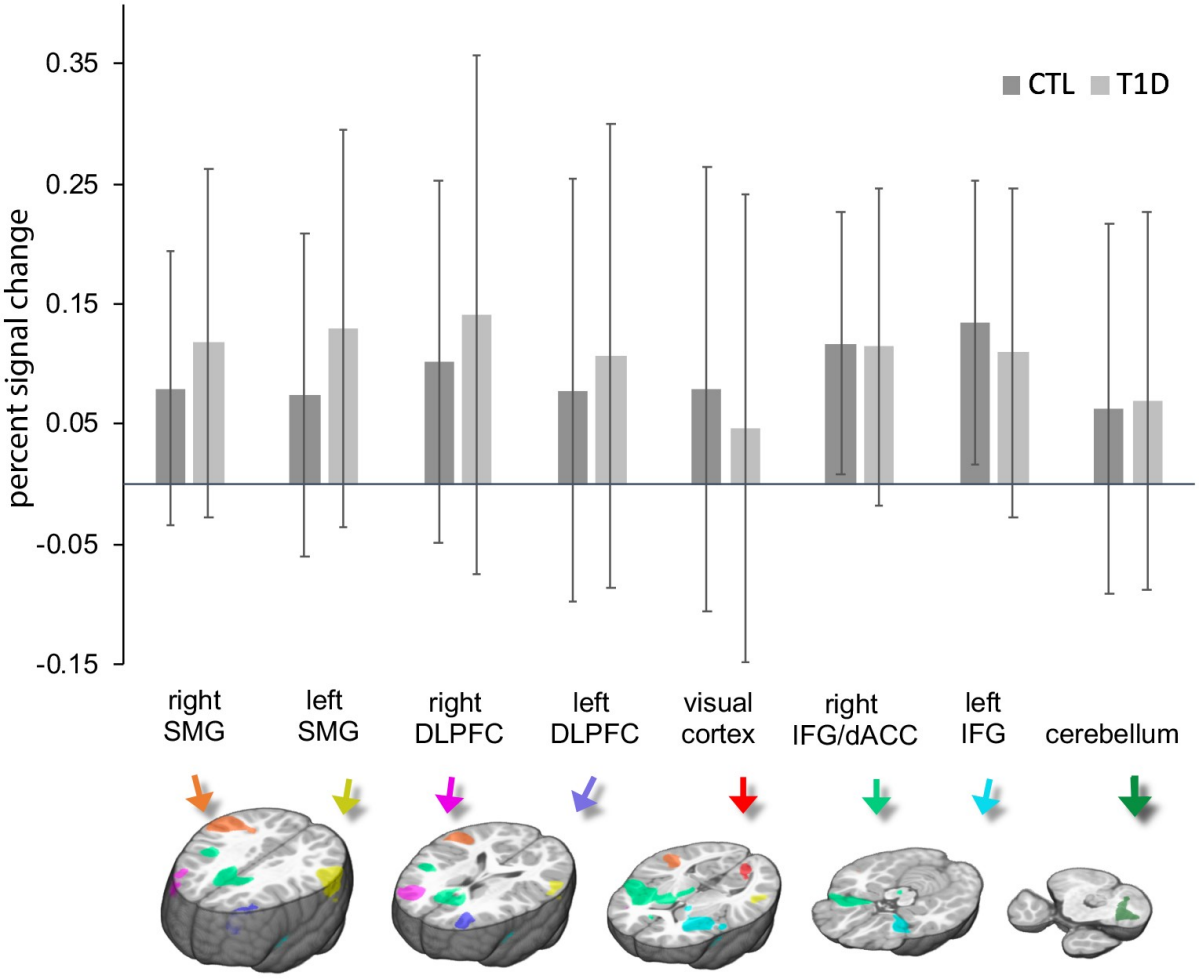
Mean activation plotted separately for each group and for each region of interest during the contrast of no-go > go. See text for details on creation of regions of interest. Error bars represent standard deviation. CTL, control; dACC, dorsal anterior cingulate cortex; DLPFC, dorsolateral prefrontal cortex; IFG, inferior frontal gyrus; SMG, supramarginal gyrus; T1D, type 1 diabetes.

Data-driven creation of executive control ROIs, created by running the no-go > go contrast across balanced groups, resulted in the following 8 ROIs: the left and right dorsolateral prefrontal cortex/frontal pole (MNI *x/y/z* centers of gravity = −33, 42, 23, and 36, 44, 22, respectively; *k* = 1,516 voxels and 2,394 voxels, respectively), the left and right supramarginal gyri (MNI *x/y/z* centers of gravity = −58, −46, 26, and 54, −38, 22 respectively; *k* = 2,918 voxels and 6,290 voxels, respectively), a region encompassing the left anterior insula and left inferior frontal cortex (MNI *x/y/z* center of gravity = −32, 13, −3; *k* = 4,316 voxels), a region including the right anterior insula, right inferior frontal cortex, and right dorsal anterior cingulate cortex (MNI *x/y/z* center of gravity = 21, 11, 18; *k* = 15,439 voxels), the left primary visual cortex (MNI *x/y/z* center of gravity = −10, −79, 5; *k* = 658 voxels), and the left cerebellum (MNI *x/y/z* center of gravity = −32, −56, −36; *k* = 1,067 voxels; Fig 2; Table 3). Analyses of percent signal change occurring in these areas during the contrast of no-go > go indicated a significant main effect of group (*F*[8,132] = 132.000, *p* = 0.010), indicating that the overall profile of activation across these ROIs was significantly different between the 2 groups (average percent signal change across ROIs: T1D, 0.116% ± 0.131%; control, 0.105% ± 0.104%). However, individual post hoc ROI pairwise comparisons were not significant (*F* values[1,139] < 3.570, *p*-values > 0.061).

**Table 3:** Activation in data-driven regions of interest.

Region	MNI coordinates *x*, *y*, *z*	Cluster size	Mean activation		*p*-Value
		(number of voxels)	T1D	Control	
All regions	—	—	0.12 ± 0.13	0.11 ± 0.10	0.010
Left DLPFC	−33, 42, 23	1,516	0.11 ± 0.19	0.08 ± 0.18	0.175
Right DLPFC	36, 44, 22	2,394	0.14 ± 0.22	0.10 ± 0.15	0.273
Left supramarginal gyrus	−58, −46, 26	2,918	0.12 ± 0.15	0.08 ± 0.11	0.061
Right supramarginal gyrus	54, −38, 22	6,290	0.13 ± 0.17	0.07 ± 0.13	0.111
Left anterior insula and inferior frontal cortex	−32, 13, −3	4,316	0.11 ± 0.14	0.13 ± 0.12	0.122
Right anterior insula, inferior frontal cortex, and dorsal anterior cingulate cortex	21, 11, 18	15,439	0.11 ± 0.13	0.12 ± 0.11	0.678
Left primary visual cortex	−10, −79, 5	658	0.05 ± 0.19	0.08 ± 0.18	0.175
Left cerebellum	−32, −56, −3	1,067	0.07 ± 0.16	0.06 ± 0.15	0.772

Activation values indicate raw percent signal change (mean ± standard deviation) during the contrast of no-go > go, in data-driven executive control regions of interest.

DLPFC, dorsolateral prefrontal cortex; MNI, Montreal Neurological Institute; T1D, type 1 diabetes.

### Secondary analyses: Associations between activation and clinical variables

Lower suppression of the PCC/PCu was associated in the T1D group with an earlier age of TID onset (*r* = −0.22, 95% CI −0.41 to −0.02, *p* = 0.033). After correction for multiple comparisons, no associations were observed in either the PCC/PCu or executive control regions with HbA_1c_, lifetime HbA_1c_AUC_6%_, MAGE, glucose CV, mean glucose, AUC180, AOCBelow70, mean blood glucose at scan, or history of severe hypoglycemia or DKA.

### Secondary analyses: Associations between activation and behavioral variables

Comparisons of standardized scores on the BASC-2, BRIEF, and WISC-IV indicated that cognitive and behavioral function was not significantly different between the 2 groups (Table 1). Better scores on parent-reported measures of executive functioning were associated in the T1D group with increased activation in executive control regions. Specifically, increased activation of the executive control network was correlated with lower externalizing behaviors (*r* = −0.35, 95% CI −0.52 to −0.15, *p* = 0.001) and reduced hyperactivity (*r* = −0.29, 95% CI −0.47 to −0.08, *p* = 0.006). No associations between activation and cognitive measures on BRIEF or BASC-2 were observed in the control group.

With respect to task performance variables, lower response times on correct go trials were associated with increased activation of the executive control network in the control group (*r* = −0.38, 95% CI −0.59 to −0.12, *p* = 0.004) as well as the T1D group (*r* = −0.36, 95% CI −0.53 to −0.16, *p <* 0.001). Correlations were not significantly different between the 2 groups (*Z* = 1.07, *p* = 0.285). Associations between activation and d-prime were not observed in either group.

### Exploratory associations between the PCC/PCu and executive control regions

To better understand the nature of possible associations between suppressed deactivation of the PCC/PCu and increased activation of executive control regions, correlation analyses were conducted between these measures across individuals, separately for each group. This analysis used 2 individual-specific mean percent signal change values: the average change across voxels within the PCC/PCu cluster (identified in between-group voxel-based analyses of the no-go > go contrast), and the average change occurring across voxels contained within all 8 executive control ROIs (during this same contrast). Individual-specific mean percent signal change values were adjusted for age, sex, and scan site and entered into bivariate Pearson correlations.

**Fig 3:**
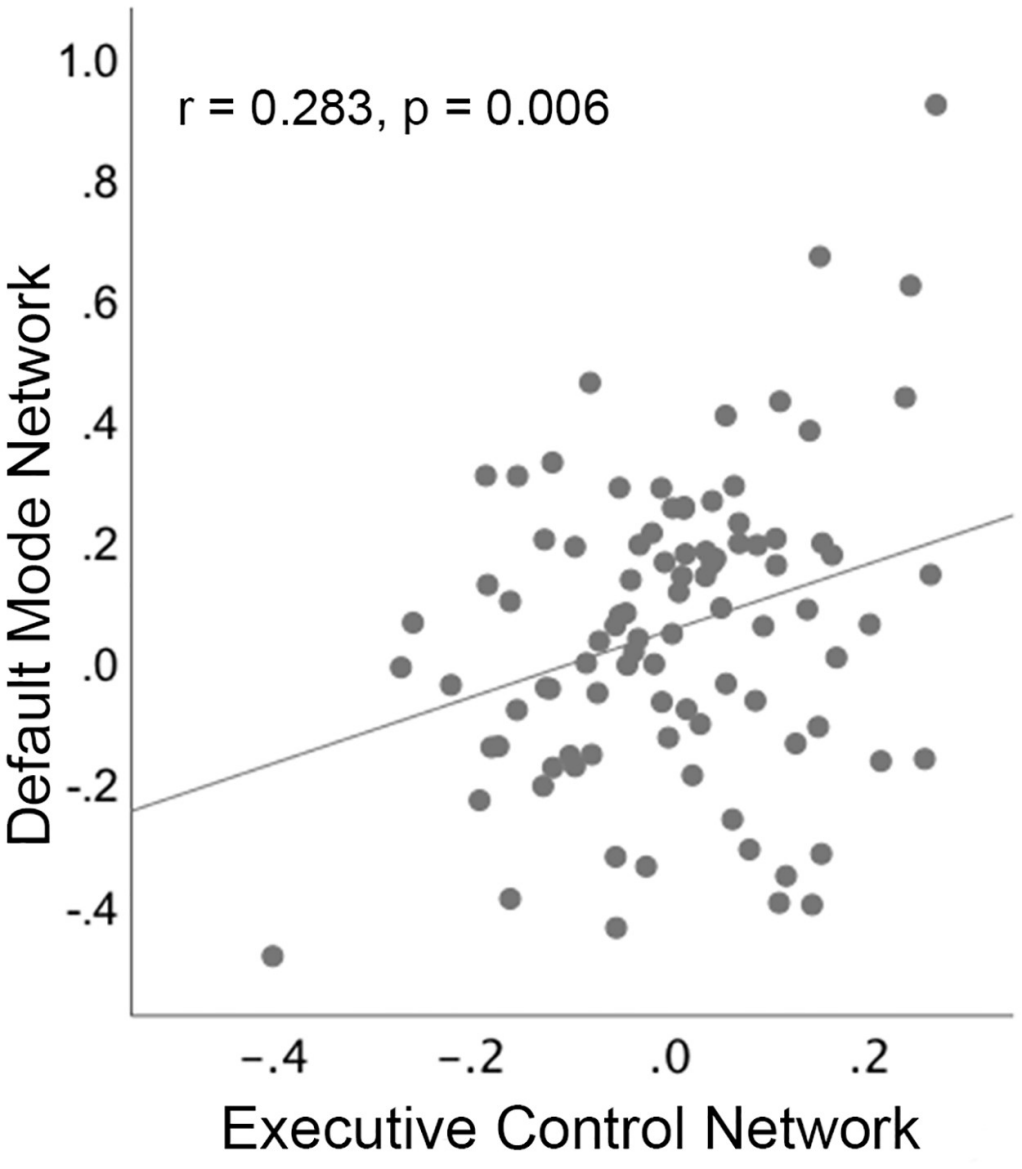
Scatterplot showing that reduced deactivation of the posterior cingulate cortex/precuneus node of the default mode network is associated with increased activation of executive control regions in children with type 1 diabetes. Activation measures represent percent signal change during no-go > go, adjusted for age, sex, and scan site.

Results of this exploratory analysis demonstrated a significant correlation between the PCC/PCu and executive control regions in the T1D group (*r* = 0.28, 95% CI 0.08 to 0.46, *p* = 0.006; Fig 3); reduced suppression of the PCC/PCu was associated with increased activation of executive control regions. This correlation was not present in the control group (*r* = 0.11, 95% CI −0.16 to 0.36, *p* = 0.423). Correlations were not significantly different between the 2 groups (*Z* = −1.18, *p* = 0.238).

## Discussion

In this large, multi-site fMRI study, we assessed activation patterns in young children with T1D as compared to age-matched non-diabetic controls while they performed the go/no-go task. Despite equivalent cognitive and behavioral performance between the 2 groups, children with T1D exhibited increased activation in executive control regions (e.g., the dorsal anterior cingulate cortex, inferior frontal gyri, cerebellum, and supramarginal gyri), as well as reduced suppression in the posterior node of the DMN (including the PCC and PCu). Secondary analyses demonstrated significant associations (*p*-values < 0.034) between activation and both behavior and disease course variables. Moreover, exploratory analyses indicated a significant relation between the DMN and executive control regions. Specifically, worse suppression of the DMN in children with T1D was associated with hyperactivation of task-positive regions underlying attentional and executive control. These data support a neural model whereby compensatory increases in activation of executive control networks may transiently counteract T1D-associated abnormalities in brain structure and/or default mode functioning, in order to facilitate normative cognitive and behavioral function.

Our findings are consistent with fMRI studies of adults with T1D [19–21], as well as with a previous investigation of RSFC that sampled from a partially overlapping, though considerably smaller, cohort of children with T1D [24]. In this prior study, T1D-associated increases in connectivity were observed within areas having known structural deficit as well as within the dorsal attention network. The dorsal attention network supports sustained attention to external stimuli, and shares significant spatial overlap with the areas identified in the current study as showing increased activation in the T1D group. Similar to this prior RSFC study, although the 2 groups in the current investigation did not differ with respect to overall cognitive function, the magnitude of activation in executive control regions was associated with better parent-reported measures of executive functioning in the T1D group. The patterns of T1D-associated differences in activation and T1D-specific correlations between activation and behavior in this group add support to a neural model whereby increased activation in executive control regions may facilitate near-normal levels of cognitive and behavioral functioning in T1D.

While unexpected, suppressed deactivation of the posterior DMN may provide a neural explanation for the mild cognitive deficits that have been reported in children with T1D [16–18]. The PCC and PCu represent a major hub of the DMN. These areas typically deactivate during the performance of goal-oriented cognitive tasks involving attention to external stimuli, and activate during internal, self-referential processes such as rumination and mind wandering [40]. The robust and highly reproducible pattern of activity in this network has led to the proposal that suppression of the DMN—and thus silencing of goal-irrelevant functions—is needed to attain optimal cognitive functioning. Consistent with this notion, functional neuroimaging studies find that greater deactivation of the DMN is associated with more successful performance on stimulus-driven cognitive tasks [41–43], including lower error rates and faster response times [44]. Failure to suppress the DMN is also observed in clinical populations in which cognitive impairment is a key feature [45–48]. In line with these findings, an earlier age of T1D onset was associated with impaired deactivation of the posterior DMN, suggesting that mounting pathology in this region could lead to diminished cognitive functioning in T1D over time.

Exploratory analyses conducted to explore a possible relation between activation in task-positive executive control regions and deactivation of the PCC/PCu indicated a significant correlation between these regions in the T1D (but not control) group. Similar patterns of increased task-positive activation and decreased task-negative deactivation have been noted in adults with T1D [19–21] and in studies of other clinical populations, such as individuals with mild cognitive impairment [49] and persons at risk for Alzheimer disease [50]. Our study is the first, to our knowledge, to test the relation between these networks in children with T1D. The current findings are important in suggesting that hyperactivation of executive control areas may act to offset cognitive dysfunction in T1D specifically caused by an impairment in deactivation of the DMN. Certainly, future studies that take both neural and cognitive function into consideration would be helpful in confirming this interpretation.

Some limitations of this investigation should be noted. We did not clamp blood glucose during scanning. Hence, further work is needed to independently assess the impact of acute versus chronic dysglycemia on the reported fMRI findings. In addition, whether the activation differences we observed between the T1D and control groups are due to inflammatory processes, subtle microvascular disease, the development of advanced glycation end products, or other factors cannot be ascertained in this observational study. Thus, additional research is needed to better elucidate the mechanisms involved in these findings.

In summary, despite equivalent cognitive and behavioral functioning between groups, young children with T1D exhibited increased activation in executive control regions (e.g., dorsal anterior cingulate cortex, inferior frontal gyri, cerebellum, and supramarginal gyri) during the performance of an attention-demanding task. The magnitude of these increases was significantly correlated with deficits in deactivation of the posterior node of the DMN, suggesting a putative compensatory role of brain function in T1D, whereby higher activation in task-relevant regions act both to offset T1D-related impairments in DMN function and to facilitate behavioral performance levels equivalent to those of their non-diabetic peers. Future studies that examine whether these patterns change as a function of improved glycemic control deserves further study.

## Abbreviations

AOCBelow70: area over the curve below blood glucose 70 mg/dl
AUC180: area under the curve above blood glucose 180 mg/dl
BASC-2: Behavior Assessment System for Children, Second Edition
BRIEF: Behavior Rating Inventory of Executive Function
CGM: continuous glucose monitor
CV: coefficient of variation
DKA: diabetic ketoacidosis
DMN: default mode network
fMRI: functional magnetic resonance imaging
HbA_1c_AUC_6%_: area under the curve HbA_1c_ > 6.0%
IDCC: imaging and data coordinating center
MAGE: mean amplitude of glycemic excursion
MNI: Montreal Neurological Institute
PCC: posterior cingulate cortex
PCu: precuneus
ROI: region of interest
RSFC: resting state functional connectivity
RT: response time
T1D: type 1 diabetes
WISC-IV: Wechsler Intelligence Scale for Children, Fourth Edition
